# Supplementary material for: Construction of habitat-specific training sets to achieve species-level assignment in 16S rRNA gene datasets
Source: Microbiome. 2020 May 15;8:65. doi: 10.1186/s40168-020-00841-w (PMC7291764; doi:10.1186/s40168-020-00841-w)
Supplement: Supplementary file 2 — Additional file 1. The expected effect of an uneven distribution of sequences among taxa in a training set for the Naïve Bayesian RDP Classifier. [file 40168_2020_841_MOESM1_ESM.docx]

**Additional File 1: The expected effect of an uneven distribution of sequences among taxa in a training set for the Naïve Bayesian RDP Classifier**

As illustrated in **Figure 2,** the naïve Bayesian RDP Classifier algorithm [1] works as follows. Given a training dataset that contains *N* unique sequences and in which the word *w_i_* appears *n*(*w_i_*) times, a prior is calculated so *P_i_* = (*n*(*w_i_*)+0.5)/(*N*+1). Then, for a given taxon (*T*) that contains *M* sequences and in which the word *w_i_* appears *m*(*w_i_*) times, the taxon-specific conditional probability for *w_i_* is *P*(*w_i_*|*T*) = (*m*(*w_i_*) + *P_i_*)/(*M* + 1). For a sequence (*S*) containing many words, *w_1_*,*w_2_*,*w_3_*, …,*w_j_*, the joint probability that *S* is derived from *T* is *P*(*S*|*T*) = *P*(*w_1_*|*T*)**P*(*w_2_*|*T*)**P*(*w_3_*|*T*)*…* *P*(*w_j_*|*T*). As *M* increases, the estimation of the prior improves, and the resolution (i.e., granularity) improves because now *P*(*w_i_*|*T*) = (*m*(*w_i_*) + *P_i_*)/(*M* + 1) is no longer limited to only two possible values, (1+ *P_i_*)/2 and (0+ *P_i_*)/2. More granular changes in *P*(*w_i_*|*T*) allow sequences with smaller differences to be distinguished, thus resolution improves.

When there is a disproportionally high number of sequences from a particular taxon present in the training set, would sequences get disproportionately assigned to that taxon? We observed that more sequences were classified unambiguously as the number of sequences (*M*) for each taxon in the training set increased (**Figure 3**). This is consistent with what Werner and colleagues reported [2]. To explain this, we will use a simplified example with two training sets: Set1 contains two taxa, *A* and *B*, with only one reference sequence each; while Set2 contains 90 sequences for taxon *A* and 10 sequences for taxon *B*, i.e., there is an uneven distribution of reads across taxa in Set2. In this simple example both the sequence of taxon *A* and taxon *B* contain four words. Three of the words, *w_1_*, *w_2_* and *w_3_* are shared but the fourth one is different, being *w_4_* for taxon *A* and *w_5_* for taxon *B*, as shown below:

Taxon *A* reference: *w_1_w_2_w_3_w_4_*

Taxon *B* reference: *w_1_w_2_w_3_w_5_*

Here we can see that for words shared by different taxa, the number of sequences in the training set and how well each taxon is represented have minimal effect. If we analyze the probabilities for the common words (*w_1_*, *w_2_* and *w_3_*) the two different training sets yield similar results, i.e., in both cases *P*(*w_i_*|*A*) = *P*(*w_i_*|*B*):

Set1: taxon *A* (1 sequence) + taxon *B* (1 sequence)

*P_i_* = (2 + 0.5) / (2+1) = 0.83

*P*(*w_i_*|*A*) = *P*(*w_i_*|*B*) = (1+0.83) / (1+1) = 0.915

Set2: taxon *A* (90 sequences) + taxon *B* (10 sequences)

*P_i_* = (100 + 0.5) / (100+1) ~= 1

*P*(*w_i_*|*A*) = (90 + *P_i_*) / (90+1) ~= 1

*P*(*w_i_*|*B*)= (10+ *P_i_*) / (10+1) ~= 1

In contrast, words that distinguish taxon *A* from taxon *B* (*w_4_* and *w_5_*) have a large effect, where *w_4_* is only in *A* and *w_5_* is only in *B*. As shown below, using Set2 increases the difference between *P*(*w_i_*|*A*) and *P*(*w_i_*|*B*) and, thus, improves the ability to classify to either *A* or *B*.

Set1: taxon *A* (1 sequence) + taxon *B* (1 sequence)

Priors *P*_4_ = *P*_5_ = (1+0.5) / (1 + 1) = 0.75

*P*(*w_4_*|*A*) = (1+0.75) / (1+1) = 0.875

*P*(*w_4_*|*B*) = (0+0.75) / (1+1) = 0.375

*P*(*w_5_*|*A*) = (0+0.75) / (1+1) = 0.375

*P*(*w_5_*|*B*) = (1+0.75) / (1+1) = 0.875

Set2: taxon *A* (90 sequences) + taxon *B* (10 sequences)

Prior *P*_4_ = (90+0.5) / (100 +1) = 0.896

*P*(*w_4_*|*A*) = (90+0.896) / (90+1) = 0.999

*P*(*w_4_*|*B*) = (0+0.896) / (10+1) = 0.081

Prior *P*_5_ = (10+0.5)/(100+1) = 0.104

*P*(*w_5_*|*A*) = (0+0.104)/(90+1) = 0.001

*P*(*w_5_*|*B*) = (10+0.104)/(10+1) = 0.919

To be more specific, for this example, when there are two query sequences, *SA* and *SB*, their probability of belonging to taxon *A* or taxon *B* is calculated as the following:

*P*(*SA*|*A*) = *P*(*w_1_*|*A*)**P*(*w_2_*|*A*)**P*(*w_3_*|*A*)**P*(*w_4_*|*A*)

*P*(*SA*|*B*) = *P*(*w_1_*|*B*)**P*(*w_2_*|*B*)**P*(*w_3_*|*B*)**P*(*w_4_*|*B*)

*P*(*SB*|*A*) = *P*(*w_1_*|*A*)**P*(*w_2_*|*A*)**P*(*w_3_*|*A*)**P*(*w_5_*|*A*)

*P*(*SB*|*B*) = *P*(*w_1_*|*B*)**P*(*w_2_*|*B*)**P*(*w_3_*|*B*)**P*(*w_5_*|*B*)

We should be able to classify *SA* as being derived from taxon *A* because *P*(*SA*|*A*) > *P*(*SA*|*B*) and *SB* to be derived from taxon *B* because *P*(*SB*|*A*) < *P*(*SB*|*B*), and it is obvious that Set2, with 90 sequences for taxon *A* and 10 sequences for taxon *B*, provides better distinction.

For simplicity, the example discussed above assumed that all 90 sequences of taxon *A* contain *w_4_* but not *w_5_* and all 10 sequences of taxon *B* contain *w_5_* but not *w_4_*. This was only to illustrate that imbalance between the number of sequences included from each taxon does not bias the classification. This is precisely because *P*(*w_i_*|*T*) = (*m*(*w_i_*) + *P_i_*)/(*M*+1). When *m*(*w_i_*) increases, so does *M*, thus *P*(*w_i_*|*T*) should be relatively stable regardless of number of sequences collected. The only concern would be that there might be situations where the proportion of sequences in *M* containing *w_i_* does not reflect the real proportion in nature. However, we think any proportion is likely to give a better estimate than 1 or 0, which is the case when you only have one sequence for each taxon.

**References**

1. Wang Q, Garrity GM, Tiedje JM, Cole JR: **Naive Bayesian classifier for rapid assignment of rRNA sequences into the new bacterial taxonomy.** *Appl Environ Microbiol* 2007, **73:**5261-5267.

2. Werner JJ, Koren O, Hugenholtz P, DeSantis TZ, Walters WA, Caporaso JG, Angenent LT, Knight R, Ley RE: **Impact of training sets on classification of high-throughput bacterial 16s rRNA gene surveys.** *ISME J* 2012, **6:**94-103.
